# Irony as a Test of the Presupposition-Denial Account: An ERP Study

**DOI:** 10.1007/s10936-021-09795-y

**Published:** 2021-08-20

**Authors:** Ruth Filik, Joanne Ingram, Linda Moxey, Hartmut Leuthold

**Affiliations:** 1 School of Psychology, University of Nottingham, Nottingham, UK; 2 School of Education and Social Science, University of the West of Scotland, Paisley, UK; 3 School of Psychology, University of Glasgow, Glasgow, UK; 4 Psychological Institute, University of Tübingen, Tübingen, Germany

**Keywords:** Irony, Presupposition-Denial account, Event-related brain potentials (ERPs), Quantifier focus, Pronoun resolution

## Abstract

According to the Presupposition-Denial Account, complement set reference arises when focus is on the *shortfall* between the amount conveyed by a natural language quantifier and a larger, expected amount. Negative quantifiers imply a shortfall, through the denial of a presupposition, whereas positive quantifiers do not. An exception may be provided by irony. One function of irony is to highlight, through indirect negation, the shortfall between what is expected/desired, and what is observed. Thus, a positive quantifier used ironically should also lead to a shortfall and license complement set reference. Using ERPs, we examined whether reference to the complement set is more felicitous following a positive quantifier used ironically than one used non-ironically. ERPs during reading showed a smaller N400 for complement set reference following an ironic compared to a non-ironic context. The shortfall generated thorough irony is sufficient to allow focus on the complement set, supporting the Presupposition-Denial Account.

## Introduction

It has been claimed that irony serves to highlight the gap between what is expected or desirable in a situation, and what is actually observed (Giora, 1995; see also Martin, 1992). For example, by saying “What a lovely party” in the middle of a terrible party, the speaker points out that the party is in fact far from being lovely by drawing attention to the gap between the expected or desired situation expressed by the literal meaning of the utterance, and reality. In this respect, irony functions in a similar manner to the purported role of negative quantifiers proposed by Moxey (2006) and Sanford et al. (2007), in the Presupposition-Denial Account. This account claims that by using a negative quantifier such as *few*, for example, in *few people came to the party*, the speaker is not only indicating that a small number of people came to the party, but also that an expectation or desire for a larger amount has been denied. In this sense, both irony and negative quantifiers can be seen as highlighting the gap between what is expected, and what is observed. In the current paper, we report an event-related brain potential (ERP) study, in which we use this property of irony in order to test the predictions of the Presupposition-Denial Account.

The Presupposition-Denial Account was developed from the observation that different quantifiers can lead to focus on different sets of discourse entities. For example, in language production tasks, different patterns of pronominal reference are observed following positive and negative quantifiers (e.g., Moxey & Sanford, 1987, 1993; Sanford et al., 1996). Specifically, when participants are presented with materials like (1) and (3), and asked to continue with a new sentence beginning with *They…*, different patterns of anaphoric reference are observed, as shown in (2) and (4):

1. Many of the diners finished their meal.
2. They… cleaned their plates and sat back happily.
3. Not many of the diners finished their meal.
4. They… got a takeaway on the way home instead.

Sentences such as (1), containing the positive quantifier *many*, tend to be followed by continuations such as (2), in which plural anaphoric reference is to the set of entities for which the predicate is true, in this case, the diners who did finish their meal. This set of entities is known as the *reference set*. In contrast, sentences containing negative quantifiers like *not many*, as in (3), tend to be followed by anaphoric reference to the set of entities for which the predicate is false, in this case, diners that did *not* finish their meal, as in (4). This set of entities is referred to as the *complement set* (Moxey & Sanford, 1987). From this it can be seen that positive quantifiers lead to focus on the reference set, whereas negative quantifiers make the complement set more salient. This observation is supported by evidence from language comprehension studies in which processing of pronominal reference to the reference set is facilitated following a positive quantifier, compared to a negative quantifier. In contrast, reference to the complement set is easier following a negative quantifier, compared to a positive quantifier (Sanford et al., 1996, Experiment 3; Paterson et al., 1998; see also Filik et al., 2011, for evidence from ERPs). This pattern of preference has also been seen in studies using sentence acceptability paradigms (Heinat & Klingvall, 2019; Upadhyay et al., 2019).

Lexical feature-based accounts (e.g., Kibble, 1997; Nouwen, 2003) explain these differences by specifying that it is the property of monotonicity^1^ which may or may not license anaphoric reference to the complement set. When a determiner, in this case the quantifier *not many*, is monotone decreasing (or downwards entailing) in the second argument it licenses inferences from supersets to subsets (Geurts & van der Slik, 2005). For instance, if *not many people came to the party*, then *not many people came to the party early*. The polarity and monotonicity of a given determiner is often assessed through its combination with negative polarity items in declarative sentences (Sanford et al., 2007). A negative polarity item is an expression which can occur in negative, but not positive, linguistic environments. Crucially, a negative quantifier licenses the use of a negative polarity item such as *anymore* (e.g., *not many politicians are honest anymore*), whereas a positive quantifier such as *many* does not (e.g., *many politicians are honest anymore* is infelicitous). Since a positive quantifier licenses inferences from subsets to supersets, as opposed to the reverse, it is considered monotone increasing and upwards entailing. Thus, according to lexical feature-based accounts, *many* should not license complement set reference.

As an alternative to lexical feature-based accounts, Moxey (2006) and Sanford et al. (2007) developed the Presupposition-Denial Account. The central tenet of this account is that negative quantifiers can give rise to both an assertion of an amount, and an implied expectation or desire that more might have been the case. Thus, *not many of the diners finished their meal* asserts that some small number of people finished the meal, and further implies that more people may have been expected, or desired, to do so. In this respect, the quantifier *not many* is simultaneously presupposing, and denying, the higher expected amount. The difference between expectation and observation is referred to as a *shortfall*, and this shortfall set then becomes the focus of attention for the reader. According to the Presupposition-Denial Account, the shortfall between what is expected and what is observed *is* the complement set, and thus reference to the shortfall is, in effect, reference to the complement set.

Following this, in order for complement set reference to occur, the crucial issue is whether or not there is a shortfall. Under normal circumstances, positive quantifiers will not give rise to a shortfall, however, there are notable exceptions to this. Sanford et al. (2007) point out two language production studies which involve expectations being introduced that are external to the quantified statement. The first example involves strong *implicit* expectations. Specifically, Moxey et al. (2001) report a case (5) where a positive quantifier led to complement set reference 25% of the time, whereas normally there would be no references to the complement set following a positive quantifier.

(5) No less than 10/10% of the fans went to the football match. They…

Moxey et al. argued that this was because 10 fans or 10% of fans seems to be a low amount, in relation to what may be expected, or desired, based on world knowledge. This mismatch between expectation and observation is a shortfall, which results in complement set reference being licensed following a positive quantifier.

The second example involves the introduction of an *explicit* expectation. Moxey (2006) set up a situation in which a character who expected a high amount was introduced into the text. This was then followed by a positively quantified statement that asserted a low amount (such as *a small number*, or *a few*), as in (6).

(6) John expected all/none of the guests to finish their dinner. A small number/A few of them ate everything on their plates. They…

In a context where *all* is expected, the positive quantifiers *a small number* and *a few* indicate that the observed amount is smaller than the expected amount, resulting in a shortfall. Moxey found that this resulted in about 25% of continuations which referred to the complement set. In addition to these results from language production tasks, Moxey et al. (2009) monitored participants’ eye movements during reading, and found that readers made fewer regressive eye movements from a complement set reference after an expectation of *all* had been denied with the positive quantifier *a small number* than in conditions where the shortfall had not been emphasised. Thus, using explicit expectations to create a shortfall induced a shift towards complement set focus in a language production task and facilitated complement set focus in online reading, even following a positive quantifier, for which complement set reference does not normally occur (see Moxey & Filik, 2010, for similar results when the shortfall is based on desire for a higher amount, rather than expectation).

One further exceptional case in which focus can be on the shortfall following a positive quantifier has been identified: the case of irony (Filik & Moxey, 2010). Filik and Moxey postulated that if irony serves to highlight the gap between what is expected or desired and what is observed, then a positive quantifier used ironically should also lead to a shortfall. For example, if someone were to utter *I see many people have come to your party*, in a context where the party had in fact proved unpopular, they would be signalling the gap between the high expected or desired number, and the number of people who actually came, that is, the shortfall. Thus, anaphoric reference to the complement set following a positive quantifier should be possible if the quantifier is used ironically. Using eye-tracking, these authors examined whether an ironically intended positive quantifier can indicate a shortfall and render a reference to the complement set acceptable. Results showed that when either a positive or negative quantifier was used ironically, complement set and reference set references were read equally easily. In a follow up language production study, the pronoun “They” was most frequently used to refer to the complement set, as opposed to the reference set, when the positive quantifier *many* was used ironically.

In further relation to the current experiment, there are a number of studies that have used ERPs to investigate referential processes (see e.g., Barber et al., 2004; Callahan, 2008; and Nieuwland & Van Berkum, 2008) and specifically plural reference (Filik et al., 2008), as well as the processing of anaphoric reference to quantified antecedents (Filik et al., 2011; Ingram & Ferguson, 2018; see also Heinat & Klingvall, 2020, for evidence in Swedish). In addition to providing excellent temporal resolution, different ERP components are generally associated with different underlying cognitive processes, and thus can be informative regarding the nature of an effect, as well as the timing. Of particular relevance to the current study is the N400 component. The N400 typically manifests as a centroparietally distributed, negative-going deflection in the ERP with an onset around 200 ms and a peak at about 400 ms, and is generally observed in response to content words (see Kutas & Federmeier, 2011, for a review). Words that are unpredictable, or are a poor fit with context either at the sentence level (e.g., Kutas & Hillyard, 1984), discourse level (Van Berkum et al., 1999), or with our knowledge of the world (Filik & Leuthold, 2008; Hagoort et al., 2004) elicit a larger N400 than those that are a good fit or are more predictable.

The processing of quantified antecedents, and pronominal reference to the complement set has also been investigated using the N400 (Filik et al., 2011; Ingram & Ferguson, 2018). Most importantly for the present purposes, complement set reference following a positive quantifier should be experienced as being anomalous (Paterson et al., 1998), and would thus be expected to elicit a larger N400 relative to a non-anomalous control condition. In support of this assumption, Filik et al., (2011; see also Ingram & Ferguson, 2018) report that complement set reference following a positive quantifier elicited a larger negativity in the N400 time interval relative to complement set reference following a negative quantifier. The current paper builds on this previous work by examining, using the N400, another manner of inducing a shortfall, in this case by using a positive quantifier ironically.

Thus, the predictions for the current study are clear. According to the Presupposition-Denial Account, reference to the complement set should be more felicitous following a positive quantifier that is used ironically, than one that is used non-ironically, since irony creates a shortfall between what is expected/desired and what is observed, and thus should make the complement set more salient. As a result, the N400 elicited by pronominal reference to the complement set following a positive quantifier should be smaller in ironic than non-ironic contexts. By contrast, lexical feature-based accounts would predict no differences between these two conditions, since both conditions contain a positive quantifier, which should not license reference to the complement set.

Furthermore, the current study also contributes to the electrophysiological literature on the on-line processing of irony. Previous ERP studies investigating the comprehension of irony have principally concentrated on what happens during processing of the ironic phrase itself (e.g., Cornejo et al., 2007; Filik et al., 2014; Katz et al., 2004; Regel et al., 2010, 2011; 2014, but see Thompson et al., 2021), whereas here we focus on the influence of irony on the processing of subsequent text.

## Method

*Participants.* Seventy-two right-handed native English speakers (35 males, 37 females) from the Glasgow University community received an inconvenience allowance to take part. They provided informed consent before participating in the study, which adhered to the principles for EEG studies as approved by the local ethics committee at the University of Glasgow. Data from two participants were removed as a result of fewer than 50% of trials remaining following EEG preprocessing and artifact rejection, resulting in data from 70 participants (34 males, 36 females) entering the analyses.

*Materials and design.* We constructed 80 experimental materials based on those used in previous research (Filik & Moxey, 2010) (see Table 1 for an example, and the Appendix for a larger selection).

**Table 1:** Example material with critical word in bold

Non-ironic (anomalous)	The milk inspector looked at the high yield recorded on the milk chart. “I see many of your cows were productive this year”, he said. “They have been **ill**,” replied the farmer.
Ironic	The milk inspector looked at the low yield recorded on the milk chart. “I see many of your cows were productive this year”, he said. “They have been **ill**,” replied the farmer.

The first sentence of each material was a context sentence which would necessitate the subsequent quantified statement to be interpreted either literally, or ironically. The second sentence comprised a verbal comment containing the positive quantifier *many*. The target sentence contained a reply to this comment, and always referred to the complement set of entities mentioned in the first comment. The target sentence was always disambiguated as referring to the complement set on the final word in the verbal comment. Thus, the experiment employed a one factor, *ironic* versus *non-ironic* design. The content of the target sentence preceding and following the critical word was identical across experimental conditions.

Items were arranged in two different stimulus presentation lists. Each item appeared in only one of its two possible conditions in a given list, but appeared in both conditions over the two lists. A given list comprised 40 materials in each of the two conditions. Thus, each participant viewed 80 experimental items, 40 in each condition. Each file also included 240 filler items. Eighty of the filler items contained quantified phrases followed by a target sentence that contained plural anaphoric reference to the reference set. Thus, participants were not able to anticipate that experimental items would always be resolved to the complement set. Eighty of the fillers described two characters having a conversation, but did not include a quantified statement (e.g., *Andrew and Paul were discussing why the boss was in a bad mood. “I didn't get the report finished on time”, said Paul*.), and the final 80 fillers did not include either a quantified statement, or any social interaction between characters (e.g., *Weather conditions were getting considerably worse on the motorway. It was sensible to turn back*.). Experimental and filler items were presented in a fixed pseudorandom order, such that no more than two experimental items appeared in a row.

Participants were tested in an electrically shielded booth with ambient light kept at a low level. Word stimuli were presented in white 16-point Helvetica font on a black background at the centre of a 21″ computer monitor at a viewing distance of 80 cm.

*Procedure.* Participants were informed about the EEG procedure and experimental task. After giving their informed consent, electrodes were applied and they were seated in a booth where they read the materials from a computer screen. There were six practice trials to familiarize them with the procedure, after which the experimenter answered any questions. There were then 10 experimental blocks, each consisting of 32 trials. Blocks were separated by a break, the duration of which was determined by the participant.

The trial sequence was as follows. Each trial started with the presentation of the first two sentences of each material (the context sentence, and the sentence containing the quantifier). Participants pressed the spacebar on a computer keyboard when they had finished reading them. A blank interval of 500 ms followed, after which a fixation cross was presented in the centre of the screen for 1000 ms. Then the word-by-word presentation of the target sentence started, during which participants were asked to maintain fixation at the centre of the screen. Each word was displayed centrally for 300 ms, with 200 ms blank intervals between successive word presentations. A break of 500 ms separated each experimental trial. For 40 participants, following approximately one in 10 of the items in each block, presentation of the critical sentence was followed by a verification statement that required a ‘true’ or ‘false’ response via a button press, in order to ensure that participants were attending to the materials, whereas the remaining participants received no secondary task.^2^ The mean correct response rate for comprehension questions was 92.3%, indicating that participants were reading for comprehension. All of the 40 participants exceeded the preset minimum of 75.0% correct responses.

*Electrophysiological Measures.* A BIOSEMI Active-Two amplifier system was used for continuous recording of electroencephalographic (EEG) activity from 72 Ag/AgCl electrodes from 10 midline positions (Fpz, AFz, Fz, FCz, Cz, CPz, Pz, POz, Oz, and Iz), 31 positions located over the left hemisphere (IO1, Fp1, AF3, AF7, F1, F3, F5, F7, F9, FC1, FC3, FC5, FT7, C1, C3, C5, M1, T7, CP1, CP3, CP5, TP7, P1, P3, P5, P7, PO3, PO7, O1, two nonstandard positions PO9’ and O9’ which were located at 33% and 66% of the M1-Iz distance, respectively), and 31 homologous positions located over the right hemisphere. EEG and EOG recordings were sampled at 256 Hz. The online reference electrode was the Biosemi Common Mode Sense (CMS) electrode (see http://www.biosemi.com/faq/cms&drl.htm for details). Off-line, EEG signals for all electrodes were recalculated to an average mastoid reference and high-pass filtered (0.1 Hz, 6 dB/oct). Horizontal and vertical electro-ocular activity (hEOG and vEOG) were calculated as follows: hEOG(t) = F10(T) − F9(t) and vEOG(t) = [(IO1(t) − P1(t)) + (IO2(t) − FP2(t))]/2. Then, (ocular) artifacts were removed and EEG data were corrected (cf. Dudschig et al., 2016) following a procedure similar to that described by Nolan et al. (2010). A predefined z-score threshold of ± 3 was used to identify outliers relating to channels, epochs, independent components, and single-channels in single-epochs. The procedure included the following successive steps that were applied to the analysis epoch of 4,000-ms total duration, starting 700 ms prior to the onset of the critical word in the target sentence.

In the first step, epochs containing extreme values in single electrodes (e.g., amplifier blockings, values larger ± 500 µV in any electrode) were removed, as were trials containing values exceeding ± 75 μV in multiple adjacent electrodes that were not related to eye movements. Secondly, z-scored variance measures were calculated for all electrodes, and noisy EEG electrodes (z-score >  ± 3) were removed if their activity was uncorrelated to EOG activity. Thirdly, this ‘cleaned’ EEG data set was subjected to a spatial independent component analysis (ICA) based on the infomax algorithm (Bell & Sejnowski, 1995). ICA components representing ocular activity (blinks and horizontal eye movements) were automatically identified using z-scored measures of the absolute correlation between the ICA component and the recorded hEOG and vEOG activity, respectively, and confirmed by visual inspection before being removed from the EEG data set. Fourthly, previously removed noisy channels were interpolated in the ICA-cleaned EEG data set using the average EEG activity of adjacent uncontaminated channels within a specified distance (4 cm, ~ 3–4 neighbours per electrode) in order to ensure a full electrode array for each participant. Finally, single trial EEG waveforms for each electrode were visually inspected, and trials still containing artifacts were removed, after which there remained on average 36 trials (out of 40; range = 21–40, median = 35 and 37) per condition.

*Data Analysis.* For artifact-free trials, the signal at each electrode site was averaged separately for each experimental condition time-locked to the onset of the critical word within the 4000-ms epochs described above. Before the measurement of ERP amplitudes, EEG and EOG activity was low-pass filtered (15 Hz, 6 dB/oct) and aligned to a 200-ms baseline prior to the onset of the critical word.

The measurement and analysis of ERPs followed the procedures established in recent N400 studies carried out in our lab (e.g., Dudschig et al., 2018). That is, mean ERP amplitudes were determined at anterior (F1, Fz, F2, FC1, FCz, FC2, C1, Cz, C2) and posterior electrodes (CP1, CPz, CP2, P1, Pz, P2, PO3, POz, PO4, and two nonstandard positions PO9’ and O9’ which were located at 33% and 66% of the M1-Iz distance, respectively) that were pooled to form one anterior and one posterior region of interest (ROI). In order to investigate the time-course of the ERP effects, we examined the ERP amplitudes not only during the N400 time interval (300–500 ms relative to the onset of the critical word) but also for a preceding time interval (200–300 ms) and one subsequent time interval (500–800 ms).

Statistical analyses were performed using Huynh–Feldt corrected repeated measures analyses of variance (ANOVA). For the analysis of ERP amplitude data, we performed an ANOVA with variables Condition (ironic vs. non-ironic) and ROI (anterior vs. posterior). Significant interactions were followed up by separate tests for anterior and posterior ROIs applying Bonferroni-correction (*α* = 0.025).

## Results

**Fig. 1:**
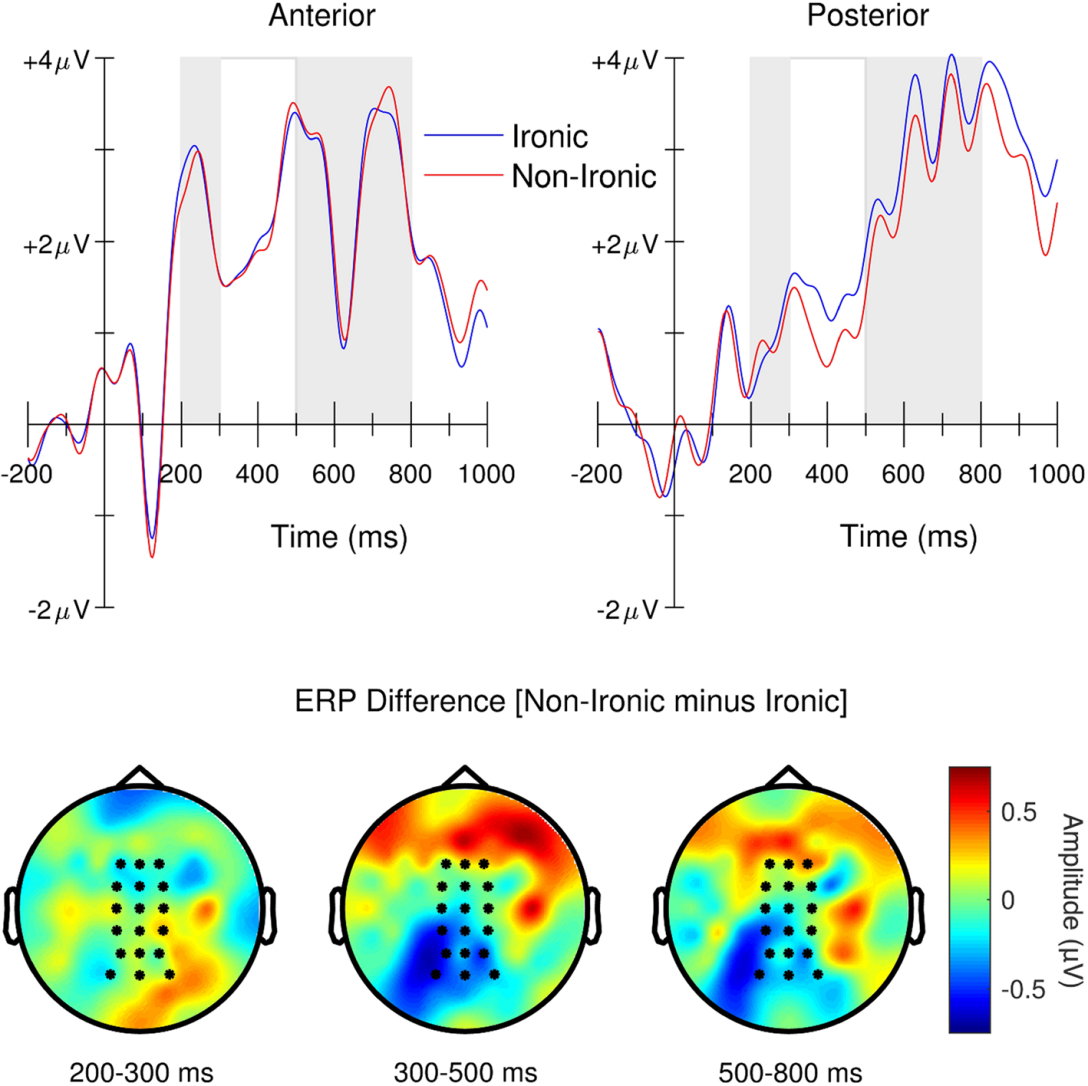
Top panel: Grand average event-related brain potentials (ERPs) at anterior and posterior ROIs following critical word onset for ironic and non-ironic conditions. Bottom panel: Topographic maps of ERP amplitudes in grand mean difference waveforms for the 200–300 ms, 300–500 ms, and 500–800 ms time intervals following critical word onset. Electrodes of anterior (F1, Fz, F2, FC1, FCz, FC2, C1, Cz, C2) and posterior ROIs (CP1, CPz, CP2, P1, Pz, P2, PO3, POz, PO4) are highlighted

Figure 1 depicts the grand average ERP waveforms triggered by the critical word in the two experimental conditions (non-ironic vs. ironic). It can be seen that the critical word triggered a series of positive-going and negative-going deflections followed by a rising late positivity, with the non-ironic condition producing a more negative-going ERP waveform than the ironic condition in the N400 time interval over the posterior ROI. The following statistical analyses corroborated this impression. It is also evident that the ERP waveform continued to be more negative-going for the non-ironic condition (cf. Figure 1). This might indicate that differences in the semantic processing of non-ironic and ironic conditions, as first indicated in the N400 time interval, were extended over time and might have occurred to different degrees at variable time points both within and between participants. Such prolonged semantic processing (see also e.g., Osterhout & Holcomb, 1992) might also have been promoted since the critical word did not appear at the sentence-final position.

*Time interval 200–300 ms*. The ANOVA with repeated measures on variables Condition (ironic vs. non-ironic) and ROI (anterior vs. posterior) revealed a main effect of ROI, *F*(1, 69) = 40.16, *p* < 0.001, *η*_*p*_^2^ = 0.37, indicating a larger positivity over the anterior than posterior ROI (2.53 vs. 0.89 μV). No other effects were significant, all *F*s < 1, *p*s > 0.56.

*N400 time interval (300–500 ms)*. Again, the ERP waveform was more positive-going over the anterior than the posterior ROI (2.15 vs. 1.45 μV), *F*(1, 69) = 15.70, *p* < 0.001, *η*_*p*_^2^ = 0.19. Crucially, there was a significant main effect of Condition (ironic vs. non-ironic = 1.80 vs. 1.59 µV), *F*(1, 69) = 4.93, *p* = 0.03, *η*_*p*_^2^ = 0.07, and a reliable Condition x ROI interaction, *F*(1, 69) = 4.45, *p* = 0.039, *η*_*p*_^2^ = 0.06. The interaction was due to a more negative-going deflection for the non-ironic than ironic sentences at the posterior ROI (1.03 vs. 1.45 µV), *F*(1, 69) = 7.98, *p* = 0.006, *η*_*p*_^2^ = 0.10, whereas this effect was absent at the anterior ROI (2.14 vs. 2.15 μV), *F*(1, 69) < 0.01, *p* = 0.95, *η*_*p*_^2^ < . 01.

*Time interval 500–800 ms*. In this time interval, the ANOVA revealed no significant effects for ROI (2.70 vs. 2.99 μV), *F*(1, 69) = 0.96, *p* = 0.33, *η*_*p*_^2^ = 0.01, Condition (2.90 vs. 2.78 μV), *F*(1, 69) = 1.12, *p* = 0.29, *η*_*p*_^2^ = 0.02, or for the interaction, *F*(1, 69) = 2.69, *p* = 0.10, *η*_*p*_^2^ = 0.04. Also, the follow-up tests did not reveal reliable condition effects over the anterior ROI (ironic vs non-ironic = 2.68 vs. 2.73 μV), *F*(1, 69) = 0.11, *p* = 0.74, *η*_*p*_^2^ < 0.01, or the posterior ROI (3.13 vs. 2.84 μV), *F*(1, 69) = 3.63, *p* = 0.06, *η*_*p*_^2^ = 0.05.

## Discussion

The key result from the current study is the significantly more negative-going ERP waveform during the N400 time interval for non-ironic than for ironic materials. This N400-like effect showed a posterior distribution and was absent in the preceding 200–300 ms time window and also not reliable in the subsequent 500–800 ms time window. Therefore, we take this N400-like effect to be related to the classical N400 effect, which is usually taken to reflect differences in semantic fit of a word with the context (see Kutas & Federmeier, 2011, for a review). It should also be mentioned that the fact that the irony-related effect was not even visible as a trend in the preceding 200–300 ms interval rules out any explanation of this N400-like effect as resulting from a potential difference in baseline activity that then propagates into the time-intervals following the onset of the critical word.^3^

In the forthcoming discussion, we will argue that the present ERP findings provide strong support for the Presupposition-Denial Account, and strong evidence against lexical feature-based accounts. The Presupposition-Denial Account states that in order for reference to the complement set to be felicitous, there must be a shortfall, since the shortfall is, essentially, the complement set. There is normally no shortfall associated with positive quantifiers such as *many*; hence reference to the complement set is perceived as being anomalous, resulting in a larger N400-like amplitude as compared to reference to the reference set (e.g., Filik et al., 2011; Ingram & Ferguson, 2018). Using irony to introduce a shortfall, however, resulted in a significantly smaller N400 in ironic compared to non-ironic conditions. Thus, this N400 effect provides convincing evidence in support of the Presupposition-Denial Account.

In contrast, the N400 results clearly do not support the predictions of lexical feature-based accounts (e.g., Kibble, 1997; Nouwen, 2003), which state that reference to the complement set is only possible following monotone decreasing quantifiers. Such accounts suggest that downwards entailing quantifiers make available for reference a set which is equivalent to the maximal set minus the reference set (i.e., *all of the farmer’s cows* minus *those cows which were productive,* in relation to the example given in Table 1). The complement set represents the difference between the maximal set and the reference set and is available for reference only under conditions where a default reference to the reference set would be infelicitous (Kibble, 1997; Nouwen, 2003). Since *many* is a positive quantifier and is monotone increasing in the second argument, complement set reference should not be possible under an account which relies solely on monotonicity. As a result, there should have been no difference between the two conditions, contrary to the present N400 effect.

A recent addition to lexical feature-based accounts (Zulaica-Hernández, 2018) highlights the potential importance of the specific proportion denoted by a quantified expression in allowing for pronominal reference to the complement set. In cases where a quantifier is monotone decreasing, the highest proportional interpretation (e.g., the largest set) does not coincide with the reference set. As the complement set then represents a greater proportion of the maximal set, it may then become salient and possibly preferable for reference. Zulaica-Hernández’s explanation of complement set reference cannot explain the present N400 results as the account remains reliant on downwards monotonicity to establish that the complement set represents a higher proportion of the maximal set than the reference set. In contrast, within the present study, the highest proportion (e.g., *cows who were unproductive* in relation to the example given in Table 1) is established through the ironic use of a monotone increasing quantifier, rather than through a monotone decreasing quantifier. Furthermore, when asking participants to provide a numerical estimate of the size of a quantified expression, Heinat and Klingvall (2019), see also Sanford et al., (1996) found no evidence that set size was a determining factor in reference. The present results add to the body of evidence which suggest complement set reference cannot be explained by current theoretical linguistic accounts. As detailed in the Presupposition-Denial Account (Moxey, 2006; Sanford et al., 2007) it is the shortfall between pre-supposed and denoted amounts, rather than the size or proportion of a set denoted by the quantified expression alone, which explains complement set reference.

The current findings are in line with previous evidence from language production studies in which the introduction of an expectation for a higher amount resulted in some references to the complement set following a positive quantifier (e.g., Moxey, 2006; Moxey et al., 2001). In addition, eye-tracking research conducted by Moxey et al. (2009) showed that emphasising a shortfall through denial of a higher expected amount led to less effortful processing of a complement set reference even for the positive quantifier *a small number* (see Moxey & Filik, 2010, for similar findings relating to a desire for a higher amount). Research conducted by Ingram and Moxey (2011) found that a shortfall emphasised by using a negative emotion to deny a high desire also led to less effortful processing of a complement set reference in comparison to conditions where a shortfall was absent. In line with findings from eye-tracking research, previous ERP studies have demonstrated that processing of a pronominal reference may be modulated by the introduction of a shortfall between what is denoted, or inferred, and what is expected or desired (Filik et al., 2011; Ingram & Ferguson, 2018). These studies used negative quantifiers or emotions to establish a shortfall. The current findings demonstrate that ERPs can also be successfully used to examine more complex referential relations, in this case, pronominal reference following a negation, or denial, generated through the ironic use of a positive expression. Thus, the current findings make an important contribution to the literature on the on-line processing of anaphoric reference to negated expressions, by using a novel manipulation of irony in order to produce a shortfall in a more indirect manner.

The present research also contributes to the ERP literature on the on-line processing of irony. Previous ERP studies investigating the comprehension of irony have mainly concentrated on what happens during processing of the ironic phrase itself (e.g., Cornejo et al., 2007; Filik et al., 2014; Katz et al., 2004; Regel et al., 2010, 2011, 2014, but see Thompson et al., 2021). Our results demonstrate that introducing irony into a discourse can also have important consequences for how subsequent text will be processed. Specifically, in this case, irony can modulate which sets of discourse entities are more salient, and hence are available for pronominal reference.

Some potential limitations of the current study should be considered. While we aimed for a simple and elegant design comparing our key conditions of interest, there are a number of further control conditions which could be informative. For example, a felicitous condition in which a (non-ironic) positively-quantified sentence was followed by anaphoric reference to the reference set would allow for assessment of whether reference to the complement set following a positive quantifier used ironically was equally felicitous. However, such a comparison would be complicated by the fact that the target words in complement set versus reference set continuations would have differed across these two conditions. A further control condition in which a (non-ironic) negative quantifier was followed by complement set reference would allow for assessment of whether a positive quantifier used ironically would make the complement set equally as salient as it would be following a negative quantifier. Although interesting, any similarities or differences between these possible additional control conditions and the conditions employed in the study would not lead to different conclusions in relation to theory, but may supply converging evidence. One final issue to consider is the potential influence of the content of the context sentence that occurred prior to the quantified sentence, specifically, whether this may additionally bolster the felicitousness of complement set reference in ironic conditions. For example, in relation to the scenarios presented in Table 1, a context in which the cows have a low milk yield may be more compatible with the critical target word “ill” than the context of a high milk yield. However, a Latent Semantic Analysis examining the association between the target word and the words/phrases that differed across the context in ironic versus non-ironic conditions indicated that the content of the context in a non-ironic condition was not more strongly associated with the target word than the content of the context in the non-ironic condition (*M*_ironic_ = 0.236, *M*_non-ironic_ = 0.232; *t*(79) = 0.285, *p* = 0.777). It therefore seems unlikely that the content of the context can explain the current ERP findings.

In conclusion, the current results indicate that introducing irony into a discourse can influence processing of subsequent text, specifically, in terms of which discourse entities are made salient for subsequent pronominal reference. This finding offers strong support for the Presupposition-Denial Account, in that using irony to create a shortfall between what is expected and what is observed can modulate the processing difficulty associated with complement set reference following a positive quantifier.

## Data Availability 

A sample of materials is provided within the manuscript. Data can be accessed by contacting the last author.

## Data Availability

All analysis procedures are standard and described in detail within the manuscript.

## Appendix

Sample of experimental materials from ERP study, with critical word in bold.

Ironic

Joe appraised the tiny gathering of fans outside the hotel.

“I see many people have come to catch a glimpse of you”, he joked.

“They probably **forgot**”, replied Justin.

Non-ironic

Joe appraised the huge crowd of fans outside the hotel.

“I see many people have come to catch a glimpse of you”, he joked.

“They probably **forgot**”, replied Justin.

Ironic

Miss Edwards was looking at the long list of ‘F’s on the exam results board.

“I see many of your students have passed”, exclaimed her colleague.

“They are now in **trouble**”, replied Miss Edwards.

Non-ironic

Miss Edwards was looking at the long list of ‘A’s on the exam results board.

“I see many of your students have passed”, exclaimed her colleague.

“They are now in **trouble**”, replied Miss Edwards.

Ironic

Aaron pointed at Bob’s empty inbox.

“I see many people have responded to your email”, he mused.

“They are probably **busy**”, replied Bob.

Non-ironic

Aaron pointed at Bob’s crowded inbox.

“I see many people have responded to your email”, he mused.

“They are probably **busy**”, replied Bob.

Ironic

Martin’s sales pitch had bombed, the air was very negative.

“I see many people liked your idea”, laughed Bill.

“They are just **jealous**”, replied Martin.

Non-ironic

Martin’s sales pitch had stunned everyone, the air was very positive.

“I see many people liked your idea”, laughed Bill.

“They are just **jealous**”, replied Martin.

Ironic

The dentist’s waiting room was pretty empty.

“I see many people are keen to see you”, giggled his nurse.

“They know I don’t use **anaesthetic**”, replied the dentist.

Non-ironic

The dentist’s waiting room was pretty crowded.

“I see many people are keen to see you”, giggled his nurse.

“They know I don’t use **anaesthetic**”, replied the dentist.

Ironic

Alan and Sara looked at the overflowing ashtrays left lying around outside the bar.

“Obviously many of the customers have given up smoking because of the new law” he laughed.

“They are completely **addicted**” she replied.

Non-ironic

Alan and Sara looked at the mostly unused ashtrays left lying around outside the bar.

“Obviously many of our customers have given up smoking because of the new law” he laughed.

“They are completely **addicted**” she replied.

Ironic

The new MP was sifting through the letters of complaint with his assistant.

“I see many people are happy with your decision”, said his assistant.

“They are never **satisfied**”, replied the minister.

Non-ironic

The new MP was sifting through the letters of support with his assistant.

“I see many people are happy with your decision”, said his assistant.

“They are never **satisfied**”, replied the minister.

Ironic

The milk inspector looked at the low yield recorded on the milk chart.

“I see many of your cows are productive this year”, he said.

“They have been **ill**,” replied the farmer.

Non-ironic

The milk inspector looked at the high yield recorded on the milk chart.

“I see many of your cows are productive this year”, he said.

“They have been **ill**,” replied the farmer.

Ironic

The manager looked at the large amount of leftover cocktail after the Christmas party.

“I see many people enjoyed your *Sex on the Beach*”, he mused to his assistant.

“They just wanted **champagne**”, replied the assistant.

Non-ironic

The manager looked at the empty cocktail jugs after the Christmas party.

“I see many people enjoyed your *Sex on the Beach*”, he mused to his assistant.

“They just wanted **champagne**”, replied the assistant.

Ironic

The sports club manager looked at the dire state of the accounts.

“Obviously many people are willing to pay our membership fee,” mused his assistant.

“They think it is **extortionate**,” replied the manager.

Non-ironic

The sports club manager looked at the healthy state of the accounts.

“Obviously many people are willing to pay our membership fee,” mused his assistant.

“They think it is **extortionate**,” replied the manager.

## Footnotes

1 Our description of the monotonicity of determiners follows that of Zulaica-Hernandez (2018) where only the increase or decrease in the extension of the second argument is considered. For a more detailed account of monotonicity see Geurts and van der Slik (2005).

2 Following standard laboratory practice, the experiment was planned to include comprehension questions for all participants. Due to a programming error and its correction after 30 participants, comprehension data were obtained only for the second subset of 40 participants. Adding the factor comprehension question (yes vs. no) to the ANOVA did not alter the result pattern of ERP amplitude analyses.

3 We performed additional ERP amplitude analyses with the baseline aligned to the − 700 to − 500 ms time interval relative to the onset of the critical word, which corresponds to the 200-ms interval before presentation of the preceding word. The analysis of ERP amplitude during the − 200 to 0 ms time interval relative to the critical word (the standard baseline period) revealed no reliable main or interaction effects involving the factor condition, all Fs < 1. Moreover, condition effects for time intervals following critical word onset replicated the ones reported for analyses using the standard baseline (− 200 to 0 ms).
